# Evaluation of an Innovative Over-the-Counter Treatment for Symptoms of Reflux Disease: Quick-Dissolving Alginate Granules

**DOI:** 10.5402/2012/950162

**Published:** 2012-12-23

**Authors:** Vicki Strugala, Peter W. Dettmar, Edward C. M. Thomas

**Affiliations:** ^1^Technostics Ltd., Daisy Building, 2nd Floor, Castle Hill Hospital, Cottingham, East Yorkshire HU16 5JQ, UK; ^2^Global Professional Relations, Reckitt Benckiser Group plc, 103-105 Bath Road, Slough, Berkshire SL1 3UH, UK

## Abstract

Traditional antacids and alginate-based reflux suppressants are OTC products commonly used to treat reflux symptoms. There has been a lack of innovation of new formulations in this therapy area despite consumers finding established products unpalatable. Here we evaluate a novel product formulation which takes the form of quick-dissolving alginate granules in single-dose sachets (Gaviscon Direct Powder (GDP)). Market research and taste evaluation confirmed that reflux sufferers considered GDP to have good flavour and taste, no chalky aftertaste and dissolved rapidly in the mouth with 68% noting so within 10 seconds. GDP was considered convenient and easy to use. The consumer-driven product development was also shown to form a strong alginate raft in standardised *in vitro* conditions that met the specifications of the BP monograph (raft strength > 7.5 g). Gastric retention of GDP and a test meal was investigated in healthy volunteers using gamma scintigraphy in comparison to Liquid Gaviscon. Both products formed an alginate raft in the stomach above the test meal and emptied after the meal. The gastric retention of the GDP product was found to be noninferior to Liquid Gaviscon. In conclusion, the innovative GDP product formed an effective raft and was well liked by consumers.

## 1. Introduction

There are a wide variety of over-the-counter (OTC) treatments that allow heartburn and indigestion sufferers to self-medicate. These take the form of simple antacids (e.g., Rennie), alginate-based reflux suppressants (e.g., Gaviscon), but also H_2_-receptor antagonists (e.g., Zantac) and proton pump inhibitors (e.g., Losec) that prevent acid secretion and are also available OTC. Here we focus on antacids and alginates only. Antacids, which primarily raise oesophageal pH and neutralise some stomach acid [1], are predominantly chewable tablets but are also available in liquids. Alginate products (which form a raft and suppress reflux) are usually suspensions of single- or double-strength alginate, but chewable tablet formats are also widely available. Antacids are often formulated for taste, convenience, and price, but alginate products have a more complex formulation that is necessary for the established efficacy of these products [2–5]. The presence of reflux symptoms (e.g., heartburn) is the biggest driver for using antacids, and there is no difference between use by men or women [6]. 

Data from the few formal studies that have been completed suggest that consumers do not report antacid/alginate formulations as being pleasurable or palatable [7–12]. These studies used wine-tasting-type methodology to evaluate palatability in terms of scores for smell, flavour, texture/mouth feel, and after taste. The studies have compared as few as 4 products [9, 11] and as many as 22 [8]. Palatability for antacids/alginates assessed in these taste evaluations was poor with even the most palatable antacid scoring only 72% [8] or 67% [12]. It can be summarised that only a minority of patients like antacids, they are tolerated by three-quarters, and the remaining 20–30% consider them unpleasant [9]. Repeatedly, the Gaviscon suspension formulations (including the US formulation, GlaxoSmithKline Consumer Healthcare, Pennsylvania, USA) do very poorly in these studies often giving the lowest palatability scores [8, 9]. There have been no studies that compare tablet formulations with liquid formulations as it isfrequently liquids only thathave been investigated.

There are clearly limitations for all of these formulations with regard palatability and resulting consumer dissatisfaction. This has important consequences for compliance with optimal treatment regimens [7, 8] for these remedies leading to suboptimal dosing. 

In a therapy area that has seen minimal innovation regarding drug formulation there is an opportunity for a new concept that consumers will appreciate in terms of taste, palatability, and portability and improve treatment compliance while remaining effective. The antacid taste evaluation studies described above interestingly showed that the most palatable products were the ones with the unusual flavours including Cherry Crème [8, 12], Lemon Twist [8], and Orange [11]. Thus flavour has been shown to be an important design factor for a new consumer-driven product development. 

Here we describe a novel product formulation that is in the form of a sachet of alginate granules that dissolve rapidly in the mouth and are enjoyable, convenient, and portable. We aimed to establish that the new formulation was as effective as a current well-accepted and validated alginate raft-forming reflux suppressant using *in vivo* raft formation studies by scintigraphy and *in vitro* raft strength testing. A detailed consumer research/taste evaluation was also carried out to appraise the new formulation in heartburn sufferers.

## 2. Methods

### 2.1. Formulations

Two formulations were used, both of which were manufactured by Reckitt Benckiser Healthcare (UK) Ltd., Dansom Lane, Hull, UK.

Gaviscon Direct Powder (GDP) was a new product development in the form of a powder/granule which dispersed in the mouth without the need for water. Each sachet contained 1.45 g of product of which active ingredients were 500 mg sodium alginate, 267 mg sodium bicarbonate, and 160 mg calcium carbonate. GDP was cool mint flavour (gamma scintigraphy study) or Tropical flavour (raft strength & taste evaluation). GDP was formulated to work in the same manner as Liquid Gaviscon to form an alginate raft on contact with the gastric contents and with the same levels of active ingredients.

Comparative studies were carried out against Liquid Gaviscon (LG) suspension (Peppermint flavour) with the equivalent dose (10 mL dose containing 500 mg sodium alginate, 267 mg sodium bicarbonate, and 160 mg calcium carbonate). 

### 2.2. Market Research

Consumer market research was carried out by Ipsos Marketing on a sample of adults who had experienced heartburn or indigestion in the past 12 months and were users of heartburn and indigestion remedies in the last 6 months. Subjects were interviewed in detail and introduced to a product concept and performed a taste evaluation of GDP. Subjects were not currently to be experiencing heartburn or indigestion symptoms or to have taken medication within 2 hours of the taste evaluation. Unless otherwise stated, the percentage agreement relates to those respondents that strongly agree or slightly agree with the statement. 

Data was compared between regular Gaviscon brand users (Reckitt Benckiser) and regular Rennie traditional antacid users (Bayer Consumer Care Division, Newbury, UK) and also between mild, infrequent heartburn sufferers and severe, frequent heartburn sufferers.

### 2.3. Raft Strength

Strength of alginate rafts was tested as previously described by Hampson et al. 2005 [13], which has been adopted as a monographed method by the British Pharmacopoeia [14]. Rafts were formed by adding a single dose of product to 150 mL of 0.1 M HCl in a 250 mL glass beaker. The raft was formed around an L-shaped stainless steel wire probe held upright throughout the 30 minute raft development phase. The wire probe was pulled vertically up through the raft at a rate of 5 mm/s using a Texture Analyser (TA-XT2, Stable Micro Systems, Godalming, Surrey, UK). The force in grams to pull the wire probe through the alginate raft was recorded. 

Raft strength of LG was evaluated by direct dosing of 10 mL of liquid product (minimum dose). Raft strength of GDP was evaluated after grinding the granules to a powder that could pass through a 1000 *μ*m sieve then agitating in 10 mL of deionised water (37°C) until it was a homogenous mixture. Raft strength testing was then carried out on the 10 mL homogenised solution of GDP.

GDP was deemed to have noninferior raft strength to LG if the mean raft strength of six replicates was greater than 7.5 g as stipulated in the monograph for Alginate Raft Forming Oral Suspensions (ARFOSs) [14].

### 2.4. Scintigraphy Study

The gastric retention of alginate rafts formed by GDP and LG and a test meal was compared in healthy volunteers using gamma scintigraphy. This was a noninferiority study that took the form of a single-centre, randomised, open-label, two-period crossover in 24 healthy male volunteers. The study was conducted in accordance to ICH GCP guidelines under the Declaration of Helsinki. All subjects provided informed written consent. The study was conducted at Simbec Scintigraphics Ltd (Merthyr Tydfil Industrial Park, Mid Glamorgan, UK), and ethical approval was obtained from South East Wales Local Research Ethics Committee. Methodology was previously described in detail in Hampson et al. 2010 [15]. In brief, the test products GDP and LG were radiolabelled with Indium-111 (^111^In), and a single dose (1.45 g GDP or 10 mL LG) was administered orally 35 minutes after the start of a standard test meal of scrambled eggs on toast with orange juice radiolabelled with 99 m Technetium (^99m^Tc). The subjects were imaged by a gamma camera (Axis dual head gamma camera, Phillips Medical Systems Ltd, Reigate, Surrey, UK) fitted with medium energy, parallel hole collimators. Anterior and posterior images of 60 seconds duration were acquired simultaneously by the gamma camera immediately after the meal is finished, 20 minutes after the start of the meal, immediately after swallowing the test product dose (35 minutes after the start of the meal), and thereafter at 15 minute intervals for up to 4 hours after administration of the test product. Images were taken simultaneously in the ^111^In and ^99m^Tc channels and stored separately on the computer for subsequent analysis.

Analysis of the scintigraphic images was performed using nuclear medicine software (Odyssey V9.4B, Philips Medical Systems Ltd.). Each image in each of the ^111^In and ^99m^Tc channels was analysed by creating three regions of interest ((1) the whole stomach, (2) upper part of the stomach and extent of product and test meal, and (3) background activity). The detected counts were corrected for background radiation and decay of the isotopes. The ^99m^Tc counts were corrected for ^111^In overlap into the ^99m^Tc channel, and the geometric mean of the anterior and posterior images was used. These counts provided representation of the gastric retention of the meal and raft in the stomach.

Percent retention of the meal and the test product (raft) at each time point both in the whole stomach and upper stomach were calculated for each subject and displayed graphically. The area under the curve (AUC) (percent retention *versus* time 0–240 min) generated AUC (test product) and AUC (meal) for the whole stomach and upper stomach. Time to half empty the test product and meal (50% retention of activity) was calculated for the whole stomach.

The primary efficacy parameter was gastric retention of the test product (AUC (test product)) in the whole stomach and was compared between the two test products (GDP and LG). Analysis of variance of the log-transformed data (with terms in the model for treatment sequence, subject within sequence, period and test product) was performed. The 95% confidence interval (CI) for the mean difference in log-transformed AUC (test product) (GDP–LG) was computed using the adjusted (least squares) means and residual standard deviation (SD). GDP was deemed to have noninferior gastric retention to LG if the detransformed 95% CI fell entirely above 0.8. 

## 3. Results

### 3.1. Consumer Market Research

**Figure 1:**
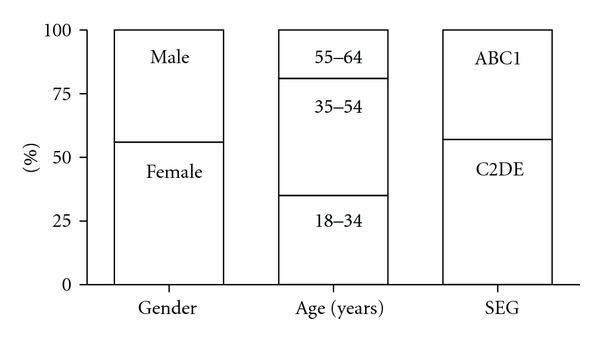
Breakdown of the demography profile of the consumer test population (*n* = 301). SEG: socioeconomic group. A: higher managerial, administrative, professional, B: intermediate managerial, administrative, professional, C1: supervisory, clerical, junior managerial, C2: skilled manual workers, D: semiskilled and unskilled manual workers, and E: casual labourers, pensioners, unemployed.

**Figure 2:**
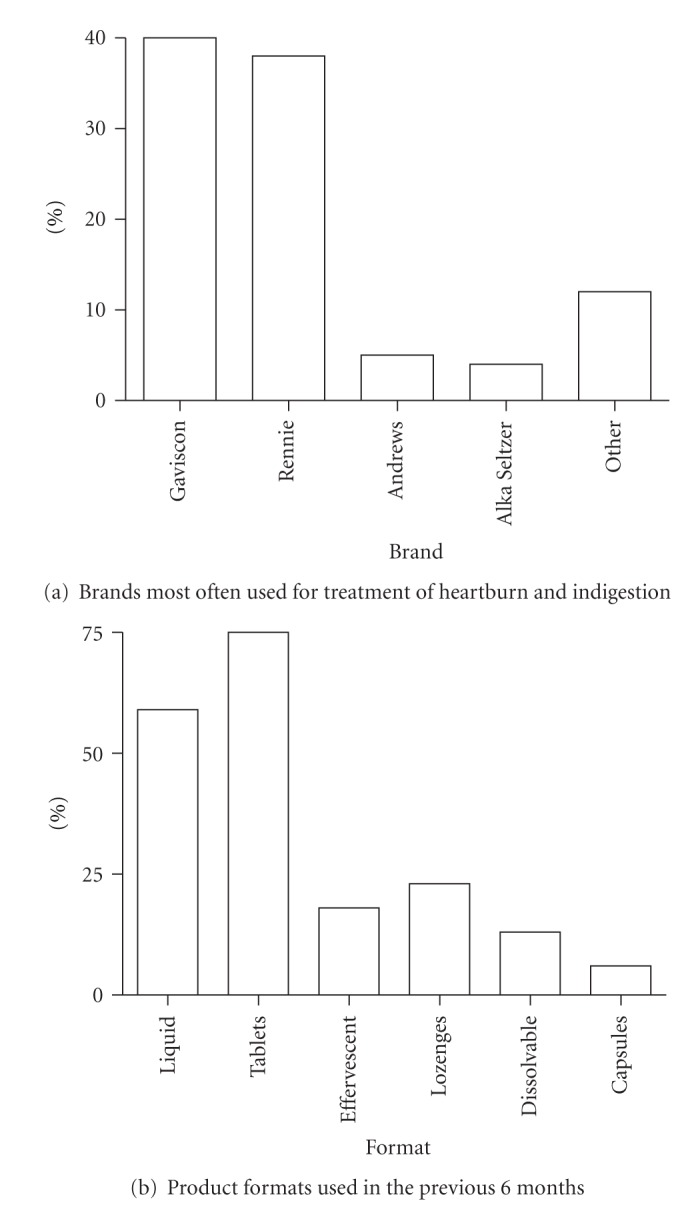
Breakdown of the product use profile of the consumer test population (*n* = 301).

The consumer market research and taste evaluation were carried out on 301 subjects (Figure 1) drawn from 8 regions of the UK (London, Southern & West, Yorkshire, Anglia, Midlands, Tyne Tees, Lancashire, Wales). The subjects most often used Gaviscon (Reckitt Benckiser) or Rennie (Bayer Consumer Care Division) as shown in Figure 2(a) and most often used liquids or chewable/swallowable tablets (Figure 2(b)).

**Figure 3:**
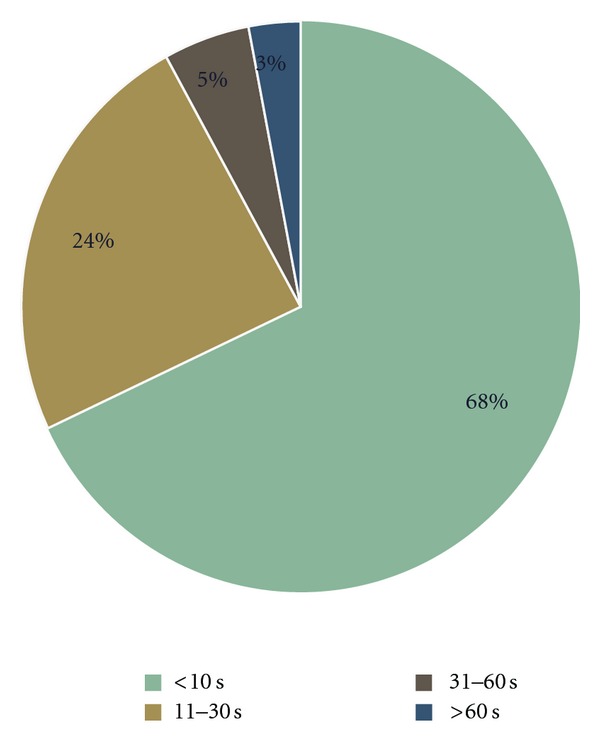
Time taken for GDP to dissolve in the mouth as assessed by a stopwatch. 68% claimed the product dissolved in the mouth within 10 seconds, 92% in 30 seconds, and 97% in 60 seconds.

Specifically related to the taste evaluation of GDP subjects were asked if GDP dissolved in the mouth. 85% of respondents agreed that the product just melted away in the mouth with 79% agreeing that it dissolved in seconds. The time taken for GDP to dissolve in the mouth was assessed using a stopwatch and is summarised in Figure 3.

Table 1 details the convenience statements and level of agreement of all the respondents as well as subanalysis of those who most often used traditional antacid (Rennie, Bayer Consumer Care Division) or Gaviscon (Reckitt Benckiser) and for the degree of suffering of heartburn and indigestion. Interestingly, consumers using traditional antacid tablets considered GDP to be hygienic to consume from. Significantly more consumers of Gaviscon described GDP as convenient, quick, and easy to use and discreet to use in public. Importantly, severe and frequent heartburn sufferers considered GDP to be discreet to use in public.

72% considered that GDP was relevant (extremely or very), 86% to be new and different (extremely or very), 92% said it was a completely new experience, and 68% of respondents said GDP was much or a little better than their usual remedy. 76% agreed that GDP was a very or quite pleasant taste (compared to 64% for their most often used remedy) with 66% agreeing that GDP had excellent or very good flavour (compared to 33% for most often used remedy).  Table 2 breaks down the response to statements about the GDP product in terms of functional benefits for all respondents and the four subgroups. It appeared that severe and frequent heartburn and indigestion sufferers had greater satisfaction with the innovative GDP product than mild and infrequent heartburn and indigestion sufferers. 

**Figure 4:**
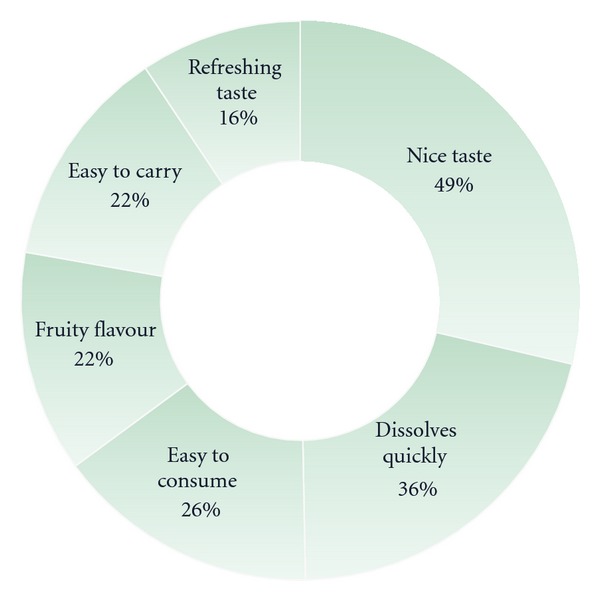
Chart to detail the six most popular spontaneous likes after the GDP taste evaluation.

The respondents were asked to provide spontaneous likes about the GDP product, and 92% provided at least one.  Figure 4 describes the six most popular spontaneous responses according to number of responses.

### 3.2. Raft Strength

Raft strength of LG and GDP comprising a dose equivalent to 500 mg of alginate was determined for six replicates. Mean raft strength of LG was 11.71 g (SD ±1.4; 95% CI 10.23–13.19). Mean raft strength for GDP was 8.30 g (SD ±2.7; 95% CI 5.45–11.16). Raft strength of LG was significantly greater than GDP (*P* = 0.0216), but since mean raft strength of GDP was >7.5 g it was deemed noninferior to LG in terms of *in vitro* raft strength and in compliance with the British Pharmacopoeia ARFOS monograph [14]. 

### 3.3. Scintigraphy Study

Forty-four subjects were screened for the study, and 24 were consented and randomised (safety population and demographic data). A single subject was ineligible for dosing on the study day, and so the efficacy evaluable population consisted of 23. All subjects were Caucasian men and a summary of demography data is provided in Table 3. 

The gastric retention of the test products (GDP or LG) are described in Table 4. There was a statistically significant difference (*P* = 0.0070) in gastric retention between GDP and LG in the whole stomach. However, the geometric mean AUC ratio (GDP/LG) was 1.186 (95% CI 1.053–1.335), and since the 95% CI falls entirely over 0.8 GDP was deemed to have noninferior gastric retention to LG.

The gastric retention of the meal (GDP or LG) is described in Table 5. There was a statistically significant difference (*P* = 0.0023) in gastric retention of meal between GDP and LG in the whole stomach. The geometric mean AUC ratio for the meal (GDP/LG) was 0.909 (95% CI 0.585–0.962) indicating that retention of the meal following GDP was significantly less than following LG.

**Figure 5:**
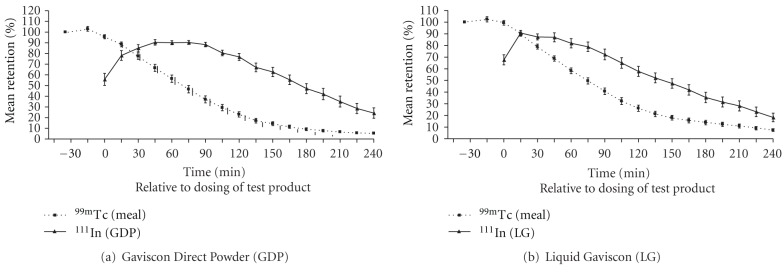
Percentage retention of ^99m^Tc (meal) and ^111^In (test product) for the whole stomach against time relative to dosing of test product (note: meal starts 35 minutes before test product dosing). Data are mean (SE) of *n* = 23.

The half emptying time indicates that the meal empties out of the stomach before the alginate raft. Comparison of gastric residence of the test product compared to the meal (geometric mean ratio AUC test product/meal) confirms that the test product remains in the stomach longer than the meal with an AUC test product/meal ratio of 1.492 (95% CI 1.349–1.652) for GDP and 1.147 (95% CI 1.019–1.290) for LG. Graphical representation of percentage retention against time for the two test products and meals is shown in Figures 5(a) and 5(b).

There were no clinically significant changes in vital signs, ECG, laboratory parameters, or physical examinations reported during the study. There were six adverse events noted by five subjects, all of which were considered unrelated or unlikely to be related to the study medication.

## 4. Discussion

GDP is a revolutionary new product formulation for the treatment of symptoms of reflux disease. The individual sachets of alginate granules give a novel dosage form within the Gaviscon line of products providing convenience and improved taste and flavour sensation while maintaining the well-documented efficacy of the alginate raft forming products. Consumer testing of the innovative granule formulation in heartburn sufferers demonstrated rapid dissolution in the mouth, a good flavour and taste profile without a chalky aftertaste. The convenience and ease of use of the new granule sachet were seen as the key advantages. The GDP product was equally well received by standard Gaviscon consumers as well as traditional antacid users and also irrespective of the extent of suffering of heartburn and indigestion, although it appeared that those with severe and frequent symptoms were more impressed with the new product formulation especially as they could safely and conveniently carry a dose with them to use at the onset of symptoms.

The *in vivo* alginate raft and meal gastric retention study in healthy volunteers concluded that gastric retention of GDP was noninferior to that of LG. Both test products formed alginate rafts that floated on the top of the stomach contents after a test meal and remained in the stomach for longer than the meal. 

Gastric residence of the meal was found to be statistically significantly reduced following GDP compared to LG. However, the half emptying time of the meal was similar between GDP and LG (100 min and 104 min, resp.), and it should be noted that based on previous alginate formulation studies the AUC (meal) values were within the expected range [16–18]. Thus the statistical significance seen between the meal retention for the two test products is unlikely to be of clinical significance. Statistically AUC (test product) for GDP was greater than LG indicating longer gastric retention, but this may be artefactual due to the slow transit through the oesophagus of a nonhydrated fvolunteers in this study.

A single dose of GDP formed an alginate raft in standardised *in vitro *conditions and exhibited raft strength greater than the level specified by the British Pharmacopoeia ARFOS monograph [14] of 7.5 g, and thus this novel granule formulation acted as expected for a raft forming alginate suspension and was noninferior to LG. 

These tests confirm that the innovative product development exhibits properties that are considered important markers in the clinical efficacy of a raft-forming alginate product; namely, that a raft is formed that is strong and floats above a meal and persists in the stomach for 4 hours; yet this new product development has a formulation design and flavour that is well liked by the consumer.

## 5. Conclusion

Gaviscon Direct Powder (GDP) is a novel formulation design in the form of individual doses of quick dissolving granules without the need for water. The product offers convenience and ease of use with rapid melting in the mouth which may provide important benefits regarding patient compliance in the treatment of symptoms of reflux. *In vitro* raft strength testing confirmed that despite the novel product formulation GDP was able to form an alginate raft which met the conditions stipulated by the British Pharmacopoeia. The *in vivo* gamma scintigraphy study in healthy volunteers further confirmed raft formation above the meal in the stomach and that GDP emptied after the meal. The raft formation and gastric retention profile were noninferior to the established Liquid Gaviscon. 

## Figures and Tables

**Table 1:** Agreement of convenience statements following the GDP taste evaluation. % agreement is those responding strongly agree or slightly agree.

Statement	% agreement				
	All *n* = 301	Gaviscon users *n* = 141	Rennie users *n* = 112	Severe and frequent sufferers	Mild and infrequent sufferers
Easier to carry and store when I am out of home	96	96	95	95	95
Makes it easy to control dosage	95	95	96	94	95
Hygienic to consume from	94	92	97*	93	94
Convenient to use when I am out of home	93	94	92	95	92
Convenient to use when I am at home	91	94^†^	88	93	91
I feel reassured storing sachets in my bag/pocket	89	89	88	92	84
Quick and easy to use	85	88^†^	79	87	81
Is discreet to use in public	82	87^†^	79	88^$^	74

^†^Significantly different to Rennie users at 95% CI.

*Significantly different to Gaviscon users at 95% CI.

^$^Significantly different to mild and infrequent sufferers at 95% CI.

**Table 2:** Agreement of benefits statement following the GDP taste evaluation. % agreement is those responding strongly agree or slightly agree.

Statement	% agreement				
	All *n* = 301	Gaviscon users *n* = 141	Rennie users *n* = 112	Severe and frequent sufferers	Mild and infrequent sufferers
It is a completely new format	91	89	90	96	90
It is like nothing I have tried before	91	92	91	95	89
It is a revolutionary product	77	77	81	85^$^	74
It just melts away in the mouth	85	85	86	90^$^	80
The product is specially formulated to dissolve instantly	79	80	79	83	79
The product dissolves in seconds	78	78	80	84	77
Has no chalky aftertaste	79	79	80	83	73
It has a refreshing taste	76	79	75	84	74
It gave me a refreshing sensation	67	70	65	77^$^	58
Has a great new sensation in the mouth	76	74	82	83^$^	71
It gave me a real soothing sensation	66	68	68	70^$^	56
It has a better taste than any HB&I medicine I have tried	72	73	72	79	68
I am delighted with the product	69	72	73	78^$^	63
It is a product I enjoyed taking	69	74	71	77^$^	61

^$^Significantly different to mild and infrequent sufferers at 95% CI.

**Table 3:** Demographic data of subjects for the scintigraphy study (*n* = 24).

Demographic parameter	Mean (SD)	Range
Age (yrs)	28.1 (9.1)	18–44
Height (m)	1.77 (0.05)	1.68–1.88
Body mass index (kg/m^2^)	24.5 (1.4)	21.9–26.5
Prestudy weight (kg)	76.2 (5.1)	67.0–90.8
Poststudy weight (kg)	76.5 (5.1)	67.5–92.2

**Table 4:** Gastric retention of test product.

Test product	AUC (test product) whole stomach	AUC (test product) upper stomach	Half emptying time
GDP	15835	13505	182 min
LG	13815	10841	151 min
	*P* = 0.0070		

**Table 5:** Gastric retention of the meal.

Test product	AUC (meal) whole stomach	AUC (meal) upper stomach	Half emptying time
GDP	10690	6463	100 min
LG	11657	7314	104 min
	*P* = 0.0023		

## References

[1] Robinson M, Rodriguez-Stanley S, Miner PB, McGuire AJ, Fung K, Ciociola AA (2002). Effects of antacid formulation on postprandial oesophageal acidity in patients with a history of episodic heartburn. *Alimentary Pharmacology and Therapeutics*.

[2] McGlashan JA, Johnstone LM, Sykes J, Strugala V, Dettmar PW (2009). The value of a liquid alginate suspension (Gaviscon Advance) in the management of laryngopharyngeal reflux. *European Archives of Oto-Rhino-Laryngology*.

[3] Chatfield S (1999). A comparison of the efficacy of the alginate preparation, Gaviscon Advance, with placebo in the treatment of gastro-oesophageal reflux disease. *Current Medical Research and Opinion*.

[4] Lindow SW, Regnéll P, Sykes J, Little S (2003). An open-label, multicentre study to assess the safety and efficacy of a novel reflux suppressant (Gaviscon Advance) in the treatment of heartburn during pregnancy. *International Journal of Clinical Practice*.

[5] Strugala V, Bassin J, Swales VS, Lindow SW, Dettmar PW, Thomas ECM (2012). Assessment of the safety and efficacy of a raft-forming alginate reflux suppressant for the treatment of heartburn during pregnancy. *ISRN Obstetrics & Gynecology*.

[6] Furu K, Straume B (1999). Use of antacids in a general population. The impact of health-related variables, lifestyle and sociodemographic characteristics. *Journal of Clinical Epidemiology*.

[7] Schneider RP, Roach AC (1976). An antacid tasting: the relative palatability of 19 liquid antacids. *Southern Medical Journal*.

[8] Temple ME, Nahata MC (2000). Comparative palatability of 22 liquid antacids. *Alimentary Pharmacology and Therapeutics*.

[9] Jacyna MR, Boyd EJS, Wormsley KG (1984). Comparative study of four antacids. *Postgraduate Medical Journal*.

[10] Blum AL (1986). Why do dyspeptic patients prefer one liquid antacid to another?. *European Journal of Clinical Investigation*.

[11] Marriott C (1983). The effect of flavour on acceptability of antacid tablets. *Journal of Clinical and Hospital Pharmacy*.

[12] Bahal-O’Mara N, Force RW, Nahata MC (1994). Palatability of 14 over-the-counter antacids. *American Pharmacy*.

[13] Hampson FC, Farndale A, Strugala V, Sykes J, Jolliffe IG, Dettmar PW (2005). Alginate rafts and their characterisation. *International Journal of Pharmaceutics*.

[14] (2007). BP: alginate raft-forming oral suspension. *British Pharmacopoeia*.

[15] Hampson FC, Jolliffe IG, Bakhtyari A (2010). Alginate-antacid combinations: raft formation and gastric retention studies. *Drug Development and Industrial Pharmacy*.

[16] Davies NM, Farr SJ, Kellaway IW, Taylor G, Thomas M (1994). A comparison of the gastric retention of alginate containing tablet formulations with and without the inclusion of excipient calcium ions. *International Journal of Pharmaceutics*.

[17] Taylor G, Warren SJ, Kellaway IW, Patel B, Little SL (1997). Gastric residence of Gaviscon Advance and Liquid Gaviscon in healthy volunteers. *Journal of Pharmacy and Pharmacology*.

[18] Hampson FC, Jolliffe IG, Bakhtyari A (2010). Alginate-antacid combinations: raft formation and gastric retention studies. *Drug Development and Industrial Pharmacy*.

